# Audit Against DVLA Guidance for New Psychiatric Patient Referrals at the Early Intervention for Psychosis Team (EIP)

**DOI:** 10.1192/bjo.2022.476

**Published:** 2022-06-20

**Authors:** Tharun Krishnan Radhakrishnan, Nismen Lathif

**Affiliations:** Mersey Care NHS Foundation Trust, Widnes, United Kingdom

## Abstract

**Aims:**

To assess the compliance of the clinicians in EIP team with DVLA guidelines. *Objectives*: To assess if there was documented evidence of: 1)Patient's diagnosis, 2)Patients’ driving status, 3)Type of vehicle driven, 4)Informing the patient that their condition may affect their ability to drive, 5)Advice regarding driving restrictions where applicable, 6)Informing the patient that they have a legal duty to inform the DVLA about their condition

**Methods:**

We selected two-thirds of the patients(n = 40) enrolled in the EIP service in the last year by consecutive sampling. We collected the data retrospectively from the clinical documentation and analysed it using excel sheets.

**Results:**

The mean age of the study sample was 34 years. 95%(n = 38) had a documented diagnosis, 67.5%(n = 27) had a documented driving status. The documentation of driving status was completed by doctors in 52%(n = 14), nurses in 26%(n = 7) and by both in 22% (n = 6). The type of vehicle driven was documented for only 33%(5) of the drivers. Among the drivers identified 33%(n = 5) had been informed that their condition might affect their driving, 67%(n = 10) had received information on driving restrictions and 47%(n = 7) had received information that they have a legal duty to inform the DVLA.

**Discussion:**

One of the reasons for the low compliance may be because another team might have documented the information at the time of referral. It is possible that the professional involved did elicit the information but didn't document the same. Healthcare professionals(HCP) have to identify, discuss and document driving-related information as advised by the DVLA. In cases where the patients’ don't follow the advice, the HCP must notify the DVLA.

**Conclusion:**

Assessment of driving history and the risks associated are critical. Awareness should be raised among the clinicians (through training and team meetings). This practice should be made an integral part of the structured initial assessments. Patients can be offered information leaflets. If successfully implemented, it will prevent unsafe driving and minimise the risk of harm for the patient and other road users.

